# Supplementary material for: Rubella Vaccine Introduction in the South African Public Vaccination Schedule: Mathematical Modelling for Decision Making
Source: Vaccines (Basel). 2020 Jul 13;8(3):383. doi: 10.3390/vaccines8030383 (PMC7565203; doi:10.3390/vaccines8030383)
Supplement: Supplementary file 1 [file vaccines-08-00383-s001.pdf]

vaccines

MDPI

Supplemental Material

Supplement 1: Model Description

We developed a discrete-time stochastic age-structured compartmental rubella transmission model for South Africa, building from previous work describing rubella dynamics [16,20]. The key feature of the model is a matrix that at every time-step defines transitions from every combination of epidemiological stage (maternally immune 'M', susceptible 'S', infected 'I', recovered 'R', and vaccinated 'V', taken to indicate the effectively vaccinated) and age group (1 month age groups up to 20 years old, then 1 year age groups up to 100 years old; 321 total age groups) to every other possible combination of epidemiological stage and age group. The discrete time-step was set to about 16 days (i.e., 24 time steps in a year), the approximate generation time of rubella. We simulated a deterministic run for each of the vaccination scenarios from year 1995 to 2050.

Epidemiological Parameters

Figure S1 displays the epidemiological transitions of the transmission model. The model is age-structured so that each epidemiological transition is age-specific, and depending on the parameter also time-specific. Here, d_a is the probability of losing maternal immunity by age class a, φ_a is the probability an individual in age class a becomes infected, r is the recovery rate, and $v_{a,t}$ is the probability an individual in age class a and time-step t is successfully vaccinated.

The duration of protection by rubella maternal antibodies ranges between 3 and 9 months; accordingly, we modelled the probability of remaining in the maternally immune epidemiological stage over age ($1-d_a$) as an exponential decay function with a constant rate of 0.95 per month [28].

The probability of infection by age, φ_a (also called the age-specific force of infection, FOI) is a function of $\mathbf{n}$(t), a vector describing the population at time t, defined as,

$$\mathbf{n}(t) = (\mathrm{M}_{1,t}, \mathrm{S}_{1,t}, \mathrm{I}_{1,t}, \mathrm{R}_{1,t}, \mathrm{V}_{1,t}, \mathrm{M}_{2,t}, \ldots \mathrm{V}_{z,t})^{\mathrm{T}}$$

according to

$$\varphi_a(\mathbf{n}(t)) = 1 - exp\,(\frac{-\sum_{\mathrm{j}} \beta_{a,j,t}\, I_{j,t}^{\gamma}}{\sum \mathbf{n}(\mathrm{t})})$$

where z is the total number of age classes (here z = 321), $\beta_{a,j,t}$ is the rate of transmission between individuals in age class a and j at time-step t, also known as the Who-Acquires-Infection-From-Whom (WAIFW) matrix, and $I_{j,t}^{\gamma}$ is the number of infected individuals in age class j and time-step t, while γ captures the non-modeled heterogeneities in age mixing and the effects of discretization of the underlying continuous process. We fix γ at 0.97 reflecting values obtained for measles in England and Wales [27] , because discrete-time models that do not incorporate this exponent result in unrealistically unstable dynamics prone to frequent extinction. Given that rubella transmission is frequency dependent, we divide the number of infected individuals in each age class by the total population size at time-step t ($\sum \mathbf{n}(t)$).

Transmission to individuals in age group a from individuals in age group j for each time-step t is defined by $\beta_{a,j,t} = \overline{\beta_{a,J,}}(1 + \alpha\, cos(2\pi t))$, where $\overline{\beta_{a,J}}$ is mean transmission from individuals in age group j to age group a, and α is a parameter controlling the magnitude of seasonal fluctuations. Previous validation of this model has shown that model results for the burden of CRS were robust to the magnitude of seasonal fluctuations [16]; we set α to 0.35 and held it constant over time [16]. Mean transmission from individuals in age class j to age class a, $\overline{\beta_{a,J}}$, was estimated by rescaling population-adjusted age-contact rates (per POLYMOD study based on diary entries [29]) to reflect the assumed basic reproductive number (R_0) of rubella. The value of R_0 used in this analysis of 7.9 and was obtained from a previously published modelling study estimating R_0 for 40 African countries [21]. We proceeded to run simulations with different estimates for R_0 in a sensitivity analysis. The highest

estimate used was an R_0 of 12 which was estimated in Ethiopia [22] and the lowest estimate estimated in Burkina Faso was 3.3 [21].

The recovery rate, *r*, is equal to 1, such that by the next time-step (or rubella generation) all infected individual will immediately move into the recovered class.

The probability an individual in age class *a* and time-step *t* is successfully vaccinated, $v_{a,t}$, depends on the assumed vaccination coverage rate assumed over time and vaccine effectiveness over age. The vaccination coverage rate ranges from 0 to 1 and is vaccination scenario specific (Table 1 in the main text). Vaccination effectiveness rate over the first 11 months of life was empirically estimated from data extracted from Boulianne et al. 1995 [29] forcing saturating at 97% and staying constant at 97% for all ages 12 months and older.

Demographic Parameters

Demographic parameters (population size, crude birth rates, and age-specific death rates) were country-specific and extracted from the United Nations World Population Prospects 2015 (cran package *wpp2015*).

The number of births per time-step *t* were estimated by multiplying the crude birth rate per time-step *t* (i.e., annual crude birth rate divided by 24 generations in a year) by the total population at time-step *t* ($\sum \mathbf{n}(t)$). Age-specific death rates as of 1995, extracted at five year age intervals, were estimated for all 321 age classes using smoothing splines and held constant over time. We assumed a constant rate of aging into the next age class (i.e., 1 divided by the length of age class *a* in years multiplied by 24).

To simulate rubella dynamics, we first needed country-specific rubella endemic populations ($\mathbf{n}(1)$). We began with fully susceptible populations based on country-specific population and age structure estimated for 1995. The one year age interval population estimates were stratified into 321 age classes using smoothing splines. In order to move beyond the transient non-seasonal outbreaks to populations representing endemic rubella, we seeded infected individuals into the population and iteratively simulated rubella dynamics for four 20-year increments assuming constant births and deaths. At the end of each 20 year cycle, we rescaled the mean transmission ($\overline{\beta_{a,J}}$) by the assumed R_0 and the population by the 1995 population and age structure, and then simulated again, four times total. The result was a country-specific population representing endemic rubella in 1995 ($\mathbf{n}(1)$). In 2015, we rescaled the population size ($\mathbf{n}(t)$) based on population total estimates for the respective year to correct for small population size differences that accumulate over time in our model.

Model Outcomes of Interest

Our model outputs the number of individuals in each age class and epidemiological stage at every time-step, allowing us to directly extract the number of rubella cases (i.e., the number of individuals in the 'I' infected epidemiological class) per age and time-step.

Age- and time-specific CRS cases were estimated by multiplying the age-specific number of susceptible individuals and probability of becoming infected over 16 week period (based on model output from each vaccination scenario), the sex ratio of the population and age-specific fertility rate (extracted from the United Nations World Population Prospects 2015), and finally the probability of CRS following rubella infection during the first 16 weeks of pregnancy (estimated 0.65 [14]).

The effective reproduction number (R_E) was estimated from the model output using the next generation method [40].

Model diagram of age-structured model.

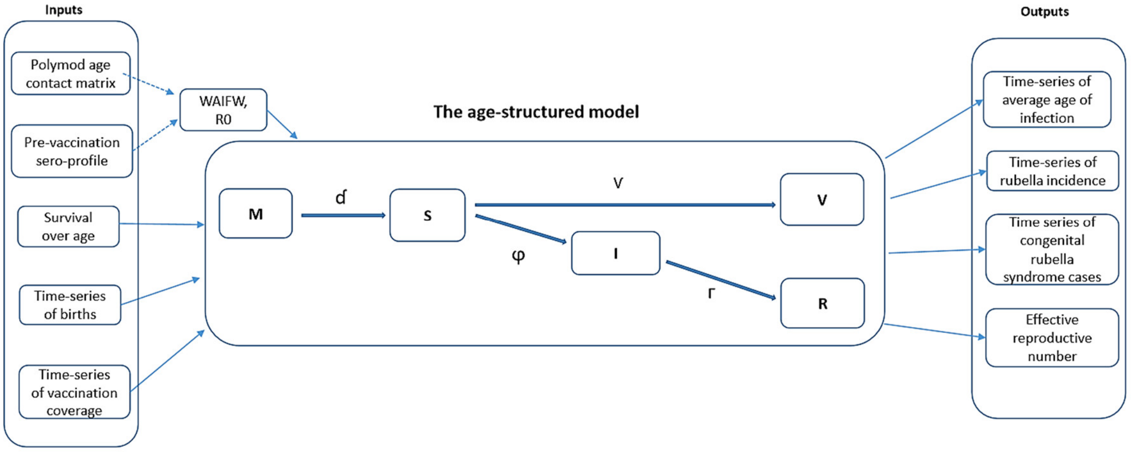

Figure S1. Relationship between data and the age-structured model. Solid lines ending in arrows indicate either data or elements inferred from data (i.e., R0, the appropriate structure of the WAIFW) that directly enter the model. Individuals in the maternal immunity (M), susceptible (S), infected (I), recovered (R) and vaccinated (V) compartments are represented with arrows representing movement between compartments: ɗ is the probability of losing maternal immunity, ϕ is the probability of becoming infected, r is the recovery rate and *v* is the probability of being vaccinated.

Supplement 2: CRS incidence over time for scenarios 2 to 6 compared to scenario 1 with extreme values of R_0

Prior to RCV introduction, the incidence of CRS when R_0 =3.3 was about three-fold that of R_0=12. A lower value of R_0 implies the rate of infection is lower. As a result, individuals are becoming exposed to the pathogen later in life. The risk of infection is therefore higher in adulthood compared to the case when R_0 is higher and this leads to an older age distribution of infected individuals and therefore a higher CRS incidence.

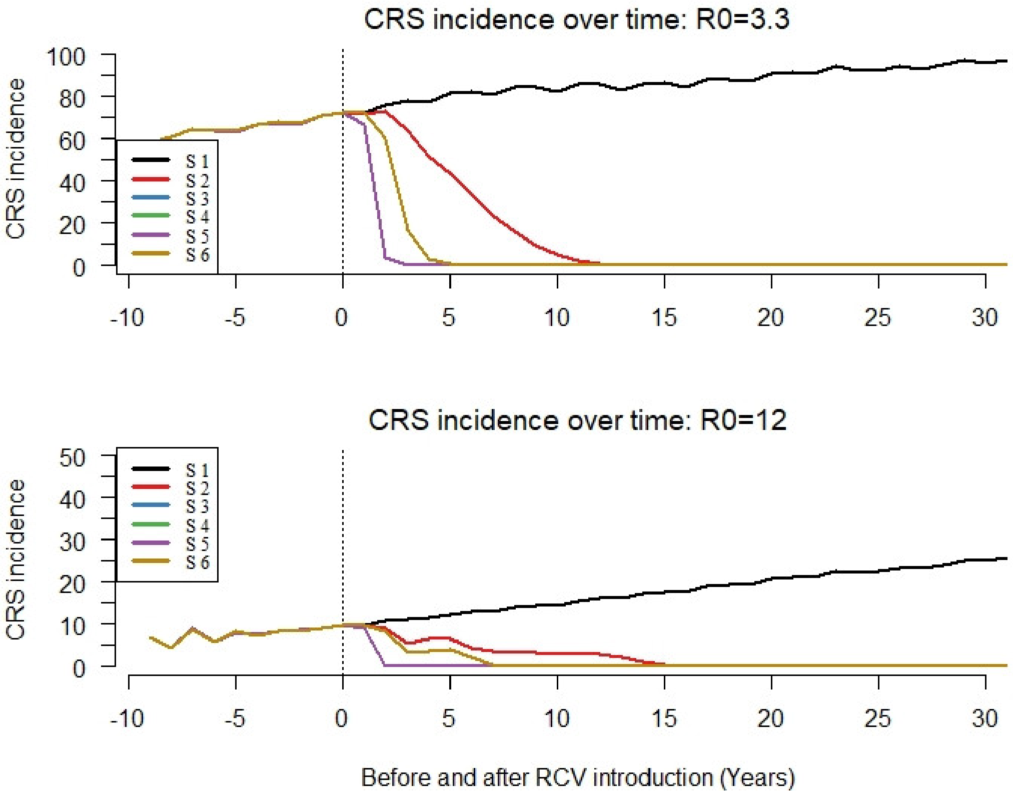

Figure S2. CRS incidence over time at 80% RCV coverage for scenarios 2 to 6 compared to scenario 1 with R0 values of 3.3 and 12. The lines for scenarios 3 and 4 overlap with that of scenario 5 so only this line is visible. The vertical dotted line represents the year of RCV introduction.

Supplement 3: CRS cases averted and DALYs averted over time for scenarios with RCV compared to no RCV

Table S3. Number of CRS cases averted and corresponding number of undiscounted DALYs averted for each scenario involving RCV introduction (2 to 6) compared to scenario 1. Estimates are shown for a range of routine vaccine coverage levels (60% through 95%) and for three time horizons: 10, 20 and 30 years.

Scenario/RCV coverage	10 years post RCV introduction		20 years post RCV introduction		30 years post RCV introduction	
	CRS averted	DALYs averted	CRS averted	DALYs averted	CRS averted	DALYs averted
Two 60%	1288	29484	2173	49759	2565	58738
Two 65%	1511	34594	3124	71543	4321	98955
Two 70%	1715	39271	4508	103240	6912	158280
Two 75%	1893	43344	5682	130122	9911	226957
Two 80%	2042	46759	6044	138408	10716	245392
Two 85%	2164	49562	6230	142658	10903	249684
Two 90%	2264	51853	6358	145601	11032	252627
Two 95%	2346	53731	6455	147814	11128	254840
Three 60%	3664	83911	7629	174711	11950	273659
Three 65%	3664	83911	7798	178574	12471	285579
Three 70%	3664	83911	7798	178584	12472	285610
Three 75%	3664	83911	7798	178584	12472	285610
Three 80%	3664	83911	7798	178584	12472	285610
Three 85%	3664	83911	7798	178584	12472	285610
Three 90%	3664	83912	7798	178585	12472	285611
Three 95%	3664	83912	7798	178585	12472	285611
Four 60%	3664	83911	7797	178560	12277	281152
Four 65%	3664	83911	7798	178584	12472	285603
Four 70%	3664	83911	7798	178584	12472	285610
Four 75%	3664	83911	7798	178584	12472	285610
Four 80%	3664	83911	7798	178584	12472	285610
Four 85%	3664	83911	7798	178584	12472	285610
Four 90%	3664	83912	7798	178585	12472	285611
Four 95%	3664	83912	7798	178585	12472	285611
Five 60%	3664	83911	7798	178584	12472	285610
Five 65%	3664	83911	7798	178584	12472	285610
Five 70%	3664	83911	7798	178584	12472	285610
Five 75%	3664	83911	7798	178584	12472	285610
Five 80%	3664	83911	7798	178584	12472	285610
Five 85%	3664	83911	7798	178584	12472	285610
Five 90%	3664	83912	7798	178585	12472	285611
Five 95%	3664	83912	7798	178585	12472	285611
Six 60%	3084	70615	7218	165288	11891	272314
Six 65%	3130	71680	7264	166353	11938	273379
Six 70%	3168	72550	7302	167223	11976	274249
Six 70%	3200	73274	7334	167947	12008	274973
Six 80%	3227	73889	7361	168562	12034	275588
Six 85%	3250	74428	7384	169101	12058	276127
Six 90%	3272	74927	7406	169600	12080	276626
Six 95%	3295	75445	7429	170118	12102	277144

Supplement 4: Change in effective reproductive number over time for all RCV coverage values.

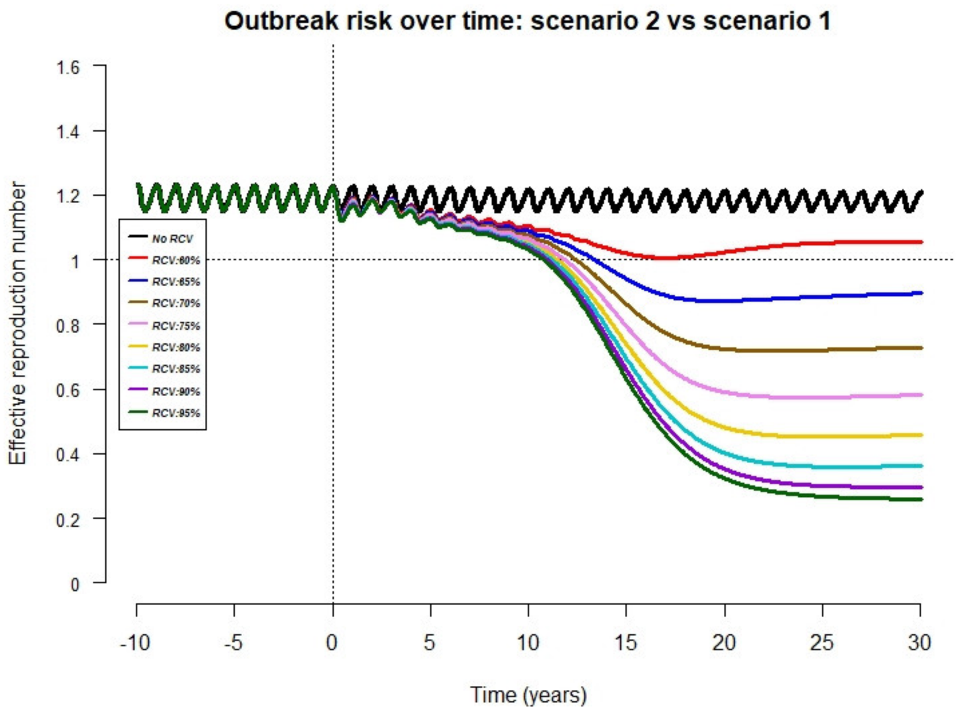

Figure S4a. Change in R_E over time for scenario 2 compared to scenario 1. While R_E never drops to values below one for 60% vaccine coverage, it takes between 11 and 14 years for R_E to drop below one with vaccine coverage levels of 65% to 95%. The slow drop in RE can be explained by the time it takes for successive vaccinated cohorts to age and achieve sufficient reduction in rubella incidence. .

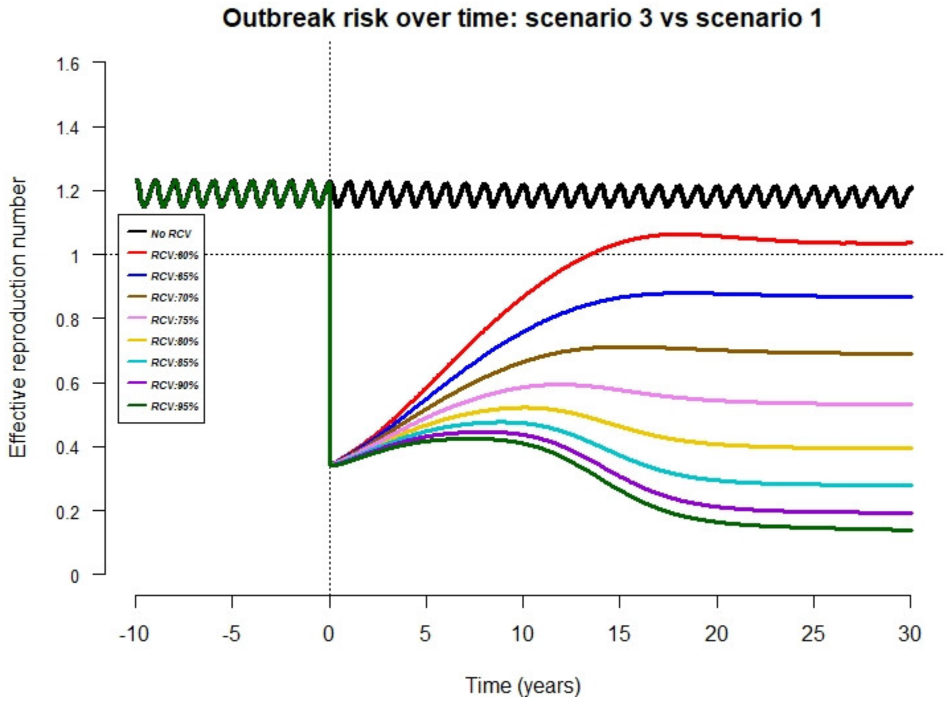

Figure S4b. Change in R_E over time for scenario 3 compared to scenario 1. Following RCV introduction, R_E immediately drops to values way below one due to the wide age range of vaccinated individuals during the initial mass campaign. There is then a progressive rise in R_E corresponding to accumulation of susceptible individuals missed during routine vaccination and the initial SIA, with

this rebound being less prominent with increasing routine vaccine coverage. R_E eventually goes above one only for 60% RCV coverage.

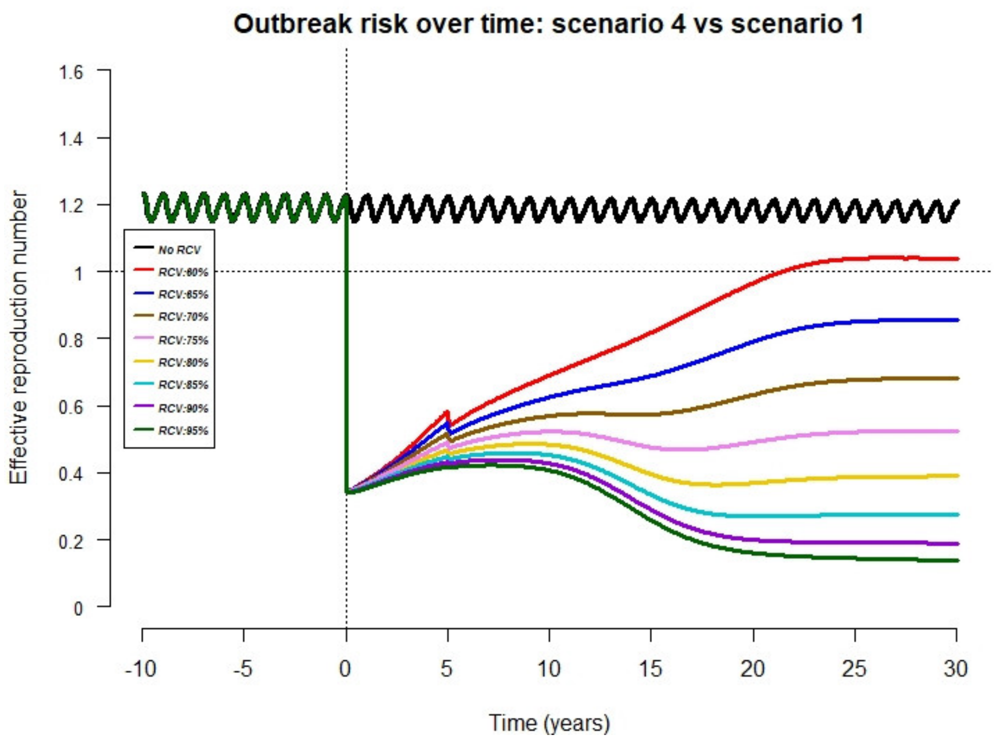

Figure S4c. Change in R_E over time for scenario 4 compared to scenario 1. Following RCV introduction, R_E immediately drops to values way below one due to the wide age range of vaccinated individuals during the initial mass campaign. There is then a progressive rise in R_E corresponding to accumulation of susceptible individuals missed during routine vaccination and the initial SIA, with this rebound being less prominent with increasing routine vaccine coverage. Following the second mass campaign 5 years after RCV introduction, R_E drops again but resumes an upward trend, eventually going above one only for 60% RCV coverage.

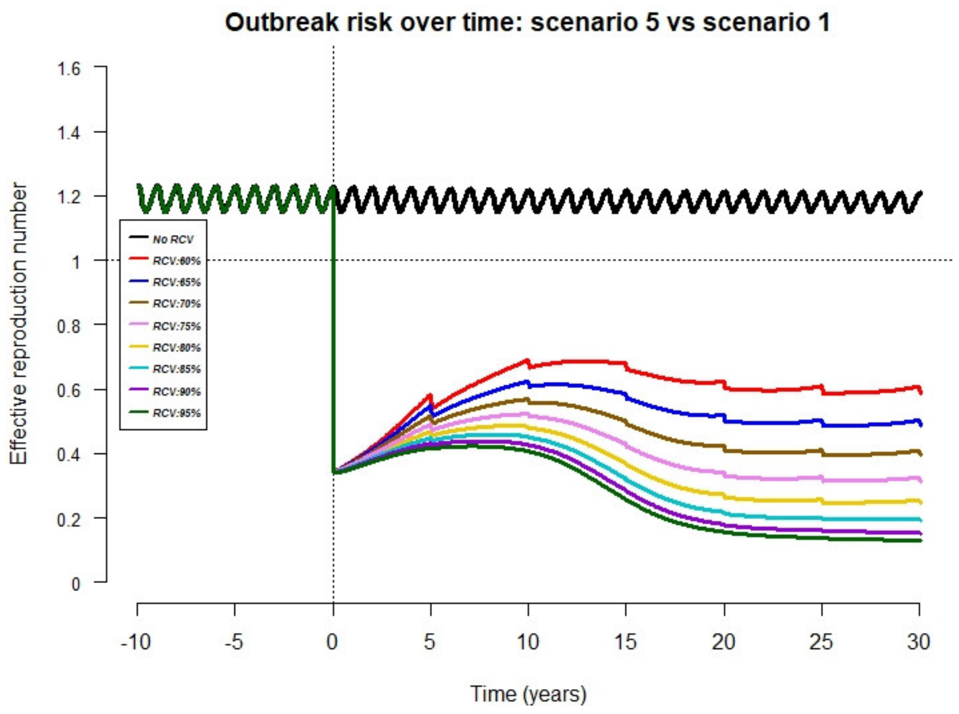

Figure S4d. Change in R_E over time for scenario 5 compared to scenario 1. Following RCV introduction, R_E immediately drops to values way below one due to the wide age range of vaccinated individuals during the initial mass campaign. There is then a progressive rise in R_E corresponding to accumulation of susceptible individuals missed during routine vaccination and the initial SIA, with this rebound being less prominent with increasing routine vaccine coverage. Following subsequent mass campaigns every 5 years, R_E drops again but resumes an upward trend. In this scenario, R_E never goes above one irrespective of RCV coverage.

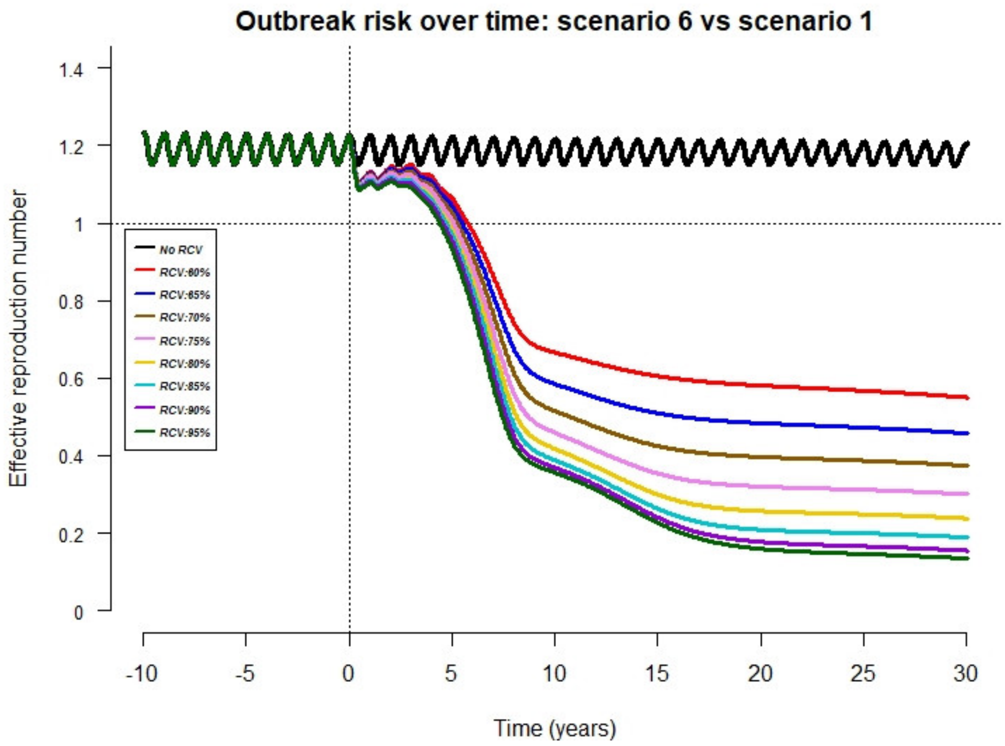

Figure S4e. Change in R_E over time for scenario 6 compared to scenario 1. It takes between 4 and 6 years for R_E to drop below one for all vaccine coverage levels with higher vaccine coverages associated with quicker decrease in R_E. The slow drop in RE can be explained by the time it takes for successive vaccinated cohorts to age and achieve sufficient reduction in rubella incidence.

Supplement 5: Change in R_E over time for scenarios 2 to 6 compared to scenario 1 with extreme values of R_0

For both values of R_0, scenarios that entail a mass campaign have an immediate drop in R_E but contrary to the lower value of R_0 (3.3), there is a rebound effect for scenarios 3 to 5 with the higher value of R_0 (12) and a slower drop in R_0 to values below one for scenarios 2 and 6. This is due to higher rubella transmission with higher R_0 values.

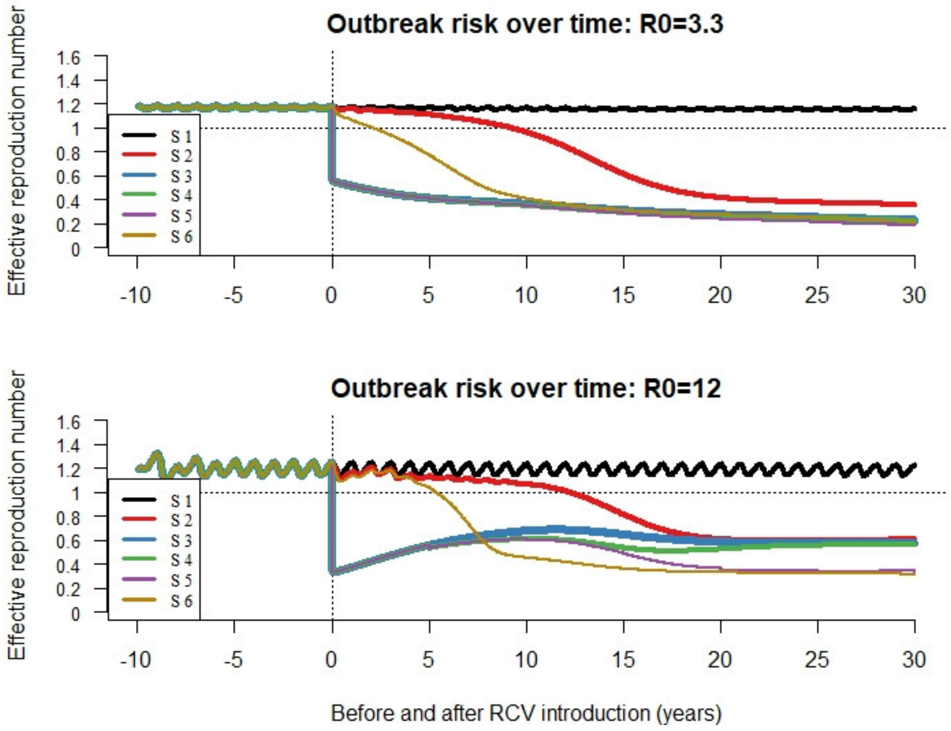

Figure S5. Change in R_E over time at 85% RCV coverage for scenarios 2 to 6 compared to scenario 1 with R0 = 12. The vertical dotted line represents the year of RCV introduction.

© 2020 by the authors. Licensee MDPI, Basel, Switzerland. This article is an open access article distributed under the terms and conditions of the Creative Commons Attribution (CC BY) license (http://creativecommons.org/licenses/by/4.0/).
